# Genetic Polymorphisms of TLR4 and MICA are Associated with Severity of Trachoma Disease in Tanzania

**DOI:** 10.16966/2470-1025.116

**Published:** 2016-06-03

**Authors:** Muneer Abbas, Noureddine Berka, Mozna Khraiwesh, Ali Ramadan, Victor Apprey, Paulette Furbert-Harris, Thomas Quinn, Hassan Brim, Georgia Dunston

**Affiliations:** 1 Department of Microbiology, Howard University, Washington, DC, USA; 2 Calgary Laboratory Services, Tissue Typing Laboratory, Canada; 3 Division of Experimental Therapeutics, Department of Drug Discovery, Walter Reed Army Institute of Research, Silver Spring, MD, USA; 4 Department of Pathology, Howard University Hospital, Washington, DC, USA; 5 Department of Community Health and Family Medicine, Howard University, Washington DC, USA; 6 International Health, School of Medicine, Johns Hopkins University, Baltimore Maryland, USA

**Keywords:** Trachoma, *Chlamydia trachomatis*, Inflammation, Trichiasis, Single nucleotide polymorphism, Toll like receptor 4, Microsatellite polymorphism, Major histocompatibility complex (MHC) class I chain related gene A, MICA

## Abstract

**Aim:**

To examine the association of TLR4 Asp299Gly and MICA exon 5 microsatellites polymorphisms with severity of trachoma in a sub-Saharan East Africa population of Tanzanian villagers.

**Methods:**

The samples were genotyped for MICA exon 5 microsatellites and the TLR4 299 A/G polymorphism by Restriction Fragment Length Polymorphism (RFLP), and GeneScan^®^, respectively. The association of TLR4 Asp299Gly and MICA exon 5 microsatellites with inflammatory trachoma (TI) and trichiasis (TI) were examined.

**Results:**

The results showed an association between TLR4 and MICA polymorphisms and trachoma disease severity, as well as with protection. TLR4 an allele was significantly associated with inflammatory trachoma (p=0.0410), while the G allele (p=0.0410) was associated with protection.

**Conclusion:**

TLR4 and MICA may modulate the risk of severity to trachoma disease by modulating the immune response to *Ct.* In addition; the increased frequency of MICA-A9 heterozygote in controls may suggest a positive selection of these alleles in adaptation to environments where *Ct* is endemic.

## Core Tip

Genetic polymorphisms in Toll like receptor 4 (TLR4) and Major histocompatibility complex (MHC) class I chain related gene (MICA) might be associated with host immunity to Trachoma disease and its subclinical phenotypes. Since their alleles vary among individuals and may confer variable disease susceptibilities, understanding the significant role that MICA and TLR4 alleles play in host inflammatory response is useful in research studies on inflammatory diseases. The data suggest that Tanzanians who carry the MICA-A9 and Asp299Gly TLR4 G alleles have lower risk to Trachoma disease.

## Introduction

Trachoma disease is characterized by repeated episodes of Gramnegative *Chlamydia trachomtis* (*Ct*) infections. The infection causes a chronic inflammatory and immune fibrogenic process leading to conjunctival scarring and blinding squeal [1]. Approximately, 84 million individuals worldwide have active infections; 1.2 million are estimated to have visual impairment, and 3% to have blindness [2,3]. The ocular disease progresses through five stages, which may overlap with increasing severity. These phases include trachomatous inflammation, follicular (TF), trachomtous inflammation, intense (TI), trachomatous scarring (TS), trachomatous trichiasis (TT), and corneal opacity (CO). According to this classification, the first two grades TF and TI represent acute infection, whereas the other three grades represent the chronic stage of the disease [4,5].

Prolonged inflammation may lead to TS and TT. Although many individuals are infected with *Ct*, only 3% will develop blinding squeal. Moreover, the majority of infections are asymptomatic and evidence has shown that disease might persist weeks after the infection has resolved itself [6]. This may indicate that host genetic factors might play an important role in modulating the inflammatory response to *Ct,* thereby determining the pathogenesis of Trachoma diseases. Candidate genes underlying the immune response to bacterial infection are ideal targets for studying their genetic polymorphisms and their association with *Ct* pathogenesis.

Major histocompatibility complex (MHC) class I chain related gene A (MICA) plays an important role as ligand for activating killer cell C-type lectin-like receptors, subfamily D (NKG2D) (natural-killer group 2, member D) receptors expressed on the surface of all CD8 αβT cells, γδT cells, and most NK cells [7]. Under normal conditions, expression of MICA molecules is restricted to intestinal and thymic epithelium [8]. When MICA is engaged by activating NKG2D, the latter functions as a signal transducer of cellular distress and triggers a range of immune effectors mechanisms including cellular cytotoxicity, cytokine secretion, and cellular proliferation [9]. There are 101 recognized human MICA alleles and 41 different MICB alleles that have been identified so far by the European Bioinformatics Institute [10,11]. The microsatellite polymorphism in exon 5 of the MICA gene consists of eight alleles based on the number of GCT/AGC triplet repeat units (alleles A4, A5, A6, A7, A8, A9, A10) and the presence of an additional nucleotide G/C insertion (allele A5.1) [12-14]. Microsatellite polymorphisms of the MICA have been shown to be associated with immunologically-mediated diseases such as the chronic systemic inflammatory disorder Behcet's disease [15].

Toll-like receptor 4 (TLR4) is a pattern recognition receptor for lipopolysaccharide (LPS) [16,17] and has been shown to induce the expression of genes involved in inflammatory responses [18,19]. Upon infection with *Ct*, the bacterial LPS is bound by LPS binding protein (LBP) and transferred to a receptor complex consisting of CD14, TLR4, and the adapter molecule MD2. This complex will initiate a signal transduction cascade leading to the release of pro-inflammatory cytokines such as tumor necrosis factor α (TNF-α) via the transcription factor nuclear factor (NFκB) [20]. Several single nucleotide polymorphisms (SNPs) in TLR4 have been reported to modulate the responsiveness to LPS by lowering the affinity to LPS [21]. Epithelial cells respond to *Ct* LPS with the nuclear translocation of NF-κB, indicating that signaling occurs through interaction with TLR4 and CD14, leading to the release of pro-inflammatory cytokines [22]. Twenty-nine SNPs have been identified in the human TLR4 gene [23]. Of these, the Asp299Gly (rs4986790) polymorphism has been shown to cause hyporesponsiveness to LPS in human alveolar macrophages and airway epithelial cell [24]. Individuals carrying the Asp299Gly TLR4 allele have lower levels of proinflammatory cytokines, acute-phase reactants, and soluble adhesion molecules, such as interleukin 6 and fibrinogen [25].

Given that inflammation and innate immunity are strongly implicated in the pathogenesis of trachoma infection, and that genetic variations in TLR4 and MICA affect the innate immune response, the aim of this study was to determine whether the TLR4 Asp299Gly and MICA microsatellite polymorphisms contribute to the susceptibility and severity of Trachoma disease in a Tanzanian population.

## Materials and Methods

Informed consent was obtained for immunogenetic studies in 1996 in accordance with the Declaration of Helsinki and was approved by the institutional review board at Johns Hopkins University The samples in this study were collected under a Johns Hopkins University approved IRB protocol for immunogenic studies in patients with Trachoma from Tanzania. We previously published first set of data from these samples [26]. All adult recruited patients gave a written consent form, while minors had their parents or guardians signed on their behalf.

### Study participants

Two subject groups were included in this study. The first was the Trichiasis Study Group (TSG), whose subjects were culled from a longitudinal study started in 1989 on the development of scarring and trichiasis in women (*n*=4932) [27]. In 1996, a subset of 186 infected and 186 uninfected women were randomly chosen from a population-based sample of women and girls aged 16 and older from 11 villages in Kongwa district, Dodoma region, Tanzania [28]. At follow-up in 1999, 74 subjects were infected and 85 were uninfected; 73% of the subjects were infected in both 1996 and 1999 with the same *chlamydia* ompA genovar. Those who had chronic infection were more likely to have had trichiasis, scarring, and active trachoma in 1996 [28,29]. Overall, there were 127 samples, and DNA was suitable for analysis in 122. Cases were women and girls with trichiasis ± inflammatory trachoma (TI) (*n*=21), and controls were women and girls with no or minimal disease (nonscarring, noninflammatory follicular trachoma, with or without infection [*n*=77]).

In the second group, the Family Trachoma Study (FTS), 15 families from one village were chosen because they had persistently infected children (probands) at three time points in a 1-year period (*n*=15). *OmpA* genotyping data indicated that this was likely persistent infection or reinfection with the same strain [30], suggesting an inability to mount a protective response. These children constituted the cases. The controls were meant to be their parents (*n*=12) and siblings (*n*=40) who had minimal or no disease (i.e., two parents had follicles, but no control had inflammation, scarring, or trichiasis).

Disease was classified using the simplified World Health Organization clinical grading scheme of the tarsal conjunctiva as follows: no trachoma (TN), ≥ 5 follicles of at least 0.5 mm each as follicular trachoma (TF), ≥ 50% of normal deep tarsal vessels obscured because of inflammatory thickening of upper tarsal conjunctiva as inflammatory trachoma (TI ± TF), and at least 1 eyelash touching the cornea or evidence of recent removal of in-turned lashes as trichiasis (TT) [31].

### Chlamydial DNA

A previously published and validated polymerase chain reaction-enzyme immunoassay for a conserved region of the MOMP-1 gene was used to detect *C. trachomatis* infection from lysed tarsal conjunctival swabs [32]. This study also describes the collection and processing of ocular samples. Every fifth PCR sample consisted of a swab inserted into PCR buffer by the sampler in the field. The contamination rate was 0.1%.

### DNA extraction

DNA was extracted as described in a previous publication [26]. DNA samples from buccal mucosa were obtained in 1999 in the field, using sterile nylon cytology brushes (Medical Packaging Corp, Camarillo CA). This method was used because peripheral blood mononuclear cells were difficult to obtain at the time, and this method has been shown to be suitable for different genetic analysis methods. The brush was gently twirled on the buccal surface, and then placed into separate envelopes and stapled to the participant's data sheet and consent form. Samples were dried in the porous envelope, which aided in the preservation of the DNA. The cytology brushes were placed in 0.6 ml of 50 mM NaOH and 0.2 mM EDTA, followed by incubation at 80°C for 10 minutes, and cooling to 25°C. The brushes were then gently agitated and removed to release DNA. The samples were neutralized with 50 μl of 1 M Tris-Cl. For longer storage, an equal volume of 90% glycerol/water was added to the lysate and preserved at -80°C.

In order to eliminate any suspended proteins and RNAs, all DNA samples were further purified using QIAamp (Qiagen Inc., Valencia CA). Briefly, 400 μl of the preserved lysate were placed in a 2 ml microcentrifuge tube containing 20 μl of Proteinase K. The tubes were then incubated at 56°C for 10 min, followed by the addition of 400 μl of ethanol (96–100%) to the samples. 700 μl of the mixture from the previous step was applied to the QIAamp Spin Column and was centrifuge at 6000 × g (8000 rpm) for 1 min. The QIAamp Spin Column was placed in a clean 2 ml collection tube and the filtrate was safely discarded. After repeating the centrifugation step, 500 μl of AW1 Buffer was added to the spin column and centrifugation was performed at 6000 × *g* (8000 rpm) for 1 min. This step was repeated with AW2 buffer. In the last step QIAamp Spin Column was placed in a clean 1.5 ml microcentrifuge tube to which 150 μl of AE Buffer was added followed by incubation at room temperature for 1 min, and then centrifuged at 6000 × *g* (8000 rpm) for 1 min. The purified DNA was stored at -20°C.

### Restriction fragment length polymorphism (RFLP)

TLR4Asp299Gly polymorphism was selected by designing primers from regions flanking the polymorphic site and distinguishing the normal and mutant alleles by size on agarose gels. The procedure was performed according to Lorenz et al. [33]. The forward primer sequence was altered to generate a restriction site in the minor allele. The following primer pair was used for TLR4 Asp299Gly, forward 5″-GATTAGCATACTTAGACT ACTACCTCCATG-3″ and reverse 5″-GATCAACTTCTGAAAAAGCATTCCCAC- 3″. The underlined bases in forward primer indicate the nucleotide altered to create *NcoI* (TLR4Asp299Gly) restriction site. Reactions were performed using the Platinum Taq^®^ PCR kit (Applied Biosystems, Foster City, CA, USA). Polymerase chain reaction(PCR) was performed in a total volume of 33 μl (20 to 60 ng of genomic DNA, 0.5 U *Taq* DNA polymerase, 10 pmol of each primer, 10 mmol of each dNTP, and 50 mmol MgCl_2_. Reactions were run on a 96-Well GeneAmp^®^ PCR System 9700 (Applied Biosystems, Foster City, CA USA) using the following conditions: 94°C for 1 min, then 30 cycles of 95°C for 30 s, 55°C for 30 s, and 72°C for 30 s). Eight microliters of the resulting PCR products were used for an overnight digest with restriction enzyme, and analyzed using 4% agarose gel electrophoresis system (BMA, Rockland, ME, USA) to determine the TLR4 allele.

### Microsatellite for MICA

For analysis of microsatellite repeat polymorphism in the TM region of the MICA gene, the following primers were described before [34]: forward MICAF: 5′-CTTTTTTTCAGGGAAAGTGC-3′ and reverse MICA5R:5′-CCTTACCATCTC- CAGAAACTGC-3′. The forward primer was labeled at the 5′end with 6-FAM (PE Biosystems, Foster City, CA). PCR reactions were carried out using the Ampli-Taq Gold^®^ PCR kit (Applied Biosystems, Foster City, CA, USA). PCR was performed in a total volume of 25 μL (5μl of 20 to 60 ng of genomic DNA, 0.5 U Taq DNA polymerase, 10 pmol of each primer, 10 mmol of each dNTP, and 25 mmol MgCl_2_. Reactions were loaded on a 96-Well GeneAmp^®^ PCR System 9700 (Applied Biosystems, Foster City, CA USA) using the following conditions: 94°C for 2 min, then 30 cycles of 94°C for 1 min, 55°C for 1 min, and 72°C for 2 min. To determine the number of triplet repeats in the TM region of the MICAgene, 1 μl of the pooled PCR products was mixed with 10 μl of formamide and denatured for 5 min at 95°C. The samples were then placed on ice for at least 5 min, after which they were loaded onto an ABI PRISM 3100 (Applied Biosystems, Foster City, CA USA). The data generated from ABI 3100 were analyzed using the GeneScan^®^ Analysis Software. Its algorithms automatically identify and size each peak relative to an internal size standard, as well as provide peak area and peak height information. The results from Gene Scan software were imported and filtered using Genotyper^®^ software to provide final results such as allele calls and automated table building.

### Statistical analysis

The statistical analyses were performed using the SAS/STAT^®^ software, version 9.1 (SAS Institute Inc., Cary, NC). Differences in subject characteristics were compared by the chi-square or Fisher's exact test for categorical values or by Student's t-test for continuous variables. Hardy Weinberg Equilibrium (HWE) was calculated using control subjects by comparing expected genotype frequencies to observed genotype frequencies using chi-square. For each SNP genotype, tests using the codominant, dominant, recessive, and log-additive genetic models were performed. Unconditional logistic regression models were used to calculate odds ratios (ORs) with 95% confidence intervals for genotypes in order to estimate the effect of the SNP presence on Trachoma risk controlling for age. Two-sided p-values ≤ 0.05 were considered as statistically significant. Participants with missing values were omitted from the analyses. Genetic modeling was performed using the statistical analysis package R (Lucent Technologies, Murray Hill, NJ).

## Results

The MICA-A9 allele was found to be statistically significant in both TI (OR=0.63 95% CI: 0.4164-0.9532, p= 0.0520) and TT (OR=0.53 95% CI: 0.3306-0.8496, p=0.0239) groups when compared to the control group (TN) (Table 1).

When the MICA-A9 genotypic frequency distribution was measured against the rest of the MIC-A alleles, we found that it was significantly associated with developing trichiasis (TT) when compared to the controls (OR=2.324 95% CI: 1.187-4.548, p=0.016; Table 2). Also, the relative risk is increased in subjects who are heterozygousity and homozygous for the MICA-A9. The results showed that the risk was three times higher when the MICA A9 allele was absent in the TT patients (OR=1.549 95% CI: 1.104-2.017, p=0.016; (Table 2) which indicates a protective role in the population.

The TLR4 A allele frequency was significantly higher in inflammatory trachoma cases (OR=6.350, 95% CI: 1.167-34.538, p=0.0410) while the TLR G allele was higher in the controls (OR=0.160 95% CI: 0.029-0.870, p=0.0410; Table 3). Allele and genotype frequencies did not differ significantly between TT patients and healthy controls, while in the TI group TLR4 A/A was significantly higher in the cases and the heterozygosity was higher in the controls (Table 4).

## Discussion

The data presented here show that genomic variation in the human MICA microsatellite in exon 5 and in TLR4 Asp299Gly are associated with the conjunctival inflammatory response due to the infection with the human pathogen *Ct.* pathogenic environment is a major determinant of the evolutionary pathway that leads to the change in the genetic makeup of the innate immune system genes. Polymorphisms in TLR4 and MICA are examples of these changes and have been related to the biology of the inflammatory responses to bacterial infections [35,36]. MICA interacts with NKG2D activating receptor expressed on CD8+ T cells, gamma delta T cells, and NK cells in humans [7]. MICA is known to trigger NK cells and co-stimulates some γδT cells and antigen-specific αβCD8+ T cells by engaging NKG2D expressed on these cells. In αβCD8 T cells, NKG2D/MIC engagement delivers a co-stimulatory signal that complements TCR-mediated antigen recognition on target cells. Allez et al. [37] provided evidence that MICA expression was significantly increased on intestinal epithelial cells isolated from patients with crohn's disease and inflammatory bowel disease, which indicates the role of MICA in the inflammatory response of stressed epithelial cells. Mei et al. [38] investigated the association of polymorphic extracellular domains of MICA alleles with genital *Ct* that cause tubal infertility in women. The data suggested that the MICA locus might modify host inflammatory response to *Ct* infection. In this study, MIC-A9 allele was found to be more frequent in the healthy subjects, which indicates a protective role. Mok et al. [39] reported on a possible protective role of MICA-A9 in the susceptibility to rheumatoid Arthritis in Korean subjects. Also, It has previously been reported that the MICA-A9 allele might confer protection from IDDM and celiac disease in the Spanish population [40,41]. This indicates that cells expressing MICA-A9 molecules might be properly recognized by γδ T cells, CD8+αβ T and NK cells, all of which are likely to have a role in overcoming Chlamydial infection and subsequently resolving Trachoma disease.

The noticeable degree of association of polymorphism that encodes structural modifications to the extracellular domain of TLR4 [24,42] with bacterial infections in different populations [43-45] provides compelling evidence for a role for this key innate immune signaling molecule in the response to Gram-negative bacterial infections. In our study, the TLR4 G allele was found to be protective (p=0.0410) and the A allele to have a predisposing in the TI phenotypes (p=0.0404 and 0.0410 respectively). Predictive modeling studies [24,46] indicate that this SNP is situated in the binding domain of the TLR4 protein and that inheritance of the SNP would interpose conformational changes that could potentially alter interaction of TLR4 with other molecules such as MD2 that is required for signaling. The TLR4 A/G genotype was found to be protective in the inflammatory trachoma when compared to the A/A homozygous genotype. Similar results were noticed in this study for MICA-A9 heterozygote that was relatively high in the Tanzanian population, and the risk increased as the MICA-A9 genotypes for heterozygote and homozygotes rates decreased in the healthy controls. The theory of heterozygote advantage [47] is ofen used to explain the genetic variation found in natural populations which might explain the protective role of MICA-A9 heterozygosity. Our results indicate that MICA-A9 and TLR4 Asp299Gly heterozygosity may confer a selective advantage in Tanzanian populations where Trachoma is endemic.

In summary, our findings suggest that MICA and TLR4 may associate with host immunity to Trachoma disease and its subclinical phenotypes. Since their alleles vary among individuals and may confer variable disease susceptibilities, understanding the significant role that MICA and TLR4 alleles play in host inflammatory response is useful in research studies on inflammatory diseases. Our results demonstrate that Tanzanians who carry theMICA-A9 and Asp299Gly TLR4 G alleles have lower risk to Trachoma disease. Further investigation can focus on the structure function relationship of these polymorphisms to mechanism of activity in regulation of the inflammatory response to *Ct* infections in Trachoma.

## Figures and Tables

**Table 1:** Association of MICA microsatellite alleles with TI and TT clinical phenotypes of Trachoma

Microsatellite allele	Cases	Controls	P-value	Odds Ratio [95% CI]
	TI			
	(104)	(348)		
A4	6	16	0.6261	1.27[0.5673-2.8431]
A5	7	10	0.0697	2.44[1.0657-5.5864]
A5.1	25	80	0.8239	1.06[0.6898-1.6289]
A6	40	121	0.4903	1.17[0.8018-1.7073]
A9	26	121	0.0520	0.63[0.4164-0.9532]
	TT			
	(82)	(370)		
A4	5	17	0.5672	1.35[0.5726-3.1830]
A5	5	12	0.2190	1.94[0.7926-4.7486]
A5.1	23	82	0.2534	1.37[0.8722-2.1519]
A6	31	130	0.6478	1.12[0.7410-1.6929]
A9	18	129	0.0239	0.53[0.3306-0.8496]

**Table 2:** Genotypic frequencies for MICA- A9 microsatellite in the TI and TT clinical phenotypes of Trachoma

MICA-A9 Genotype	Cases(n)	Controls(n)	p-value	Odds Ratio [95% CI]	Relative risk[95% CI]
	TI				
	(52)	(174)			
9:9	4	23	0.399	0.547[0.189-1.595]	0.582[0.214-1.499]
9:x	18	75	0.336	0.699[0.369-1.325]	0.803[0.521-1.174]
x:x	30	76	0.083	1.758[0.944-3.273]	1.321[0.970-1.708]
	TT				
	(41)	(217)			
9:9	1	26	0.092	0.184[0.031-1.103]	0.204[0.035-1.091]
9:x	16	109	0.233	0.634[0.323-1.244]	0.777[0.502-1.115]
x:x	24	82	0.016	2.324[1.187-4.548]	1.549[1.104-2.017]

X: Allele other than A9. Significant p value (<0.05)

**Table 3:** Association of TLR4 Asp299Gly alleles with Trachoma in Tanzania

TLR4 allele	Cases (n)	Controls (n)	P-value	Odds Ratio[95% CI]
	TI			
	(118)	(330)		
A	113	313	0.0410	6.350[1.167-34.538]
G	5	17	0.0410	0.160[0.029-0.870]
	TT			
	(100)	(348)		
A	94	336	0.2521	0.560[0.242-1.298]
G	6	12	0.2521	1.790[0.772-4.148]

**Table 4:** Association of TLR4 Asp299Gly genotypes with Trachoma in Tanzania

TLR4 Genotypes	Cases (n)	Controls (n)	p-value	Odds Ratio[95% CI]	Relative risk[95% CI]
	TI				
	(59)	(165)			
A/A	58	149	0.0464	6.230[1.131-34.313]	1.000[0.735-1.361]
A/G	1	15	0.0583	0.170[0.031-0.939]	0.223[0.033-1.50]
G/G	0	1	-	-	-
	TT				
	(50)	(174)			
A/A	44	163	0.1815	0.490[0.204-1.177]	1.000[0.691-1.448]
A/G	6	10	0.1303	2.240[0.920-5.453]	1.764[0.891-3.495]
G/G	0	1	-	-	-
